# Influence of High Myopia on Outcomes of iStent Inject W Implantation Combined with Cataract Surgery up to 24 Months of Follow-Up

**DOI:** 10.3390/jcm15031265

**Published:** 2026-02-05

**Authors:** Yuto Shiotani, Takeshi Yoshida, Sota Yoshimoto, Keigo Sugisawa, Shintaro Yasuda, Motohisa Ohno, Ryu Teramatsu, Hiroki Iemura, Yusaku Shibata, Kyoko Ohno-Matsui

**Affiliations:** 1 Department of Ophthalmology and Visual Science, Institute of Science Tokyo, Tokyo 113-8519, Japan; ytshiotani@gmail.com (Y.S.); sk2011bef@gmail.com (K.S.); moto_2495@yahoo.co.jp (M.O.); tachimiroku666@gmail.com (H.I.);; 2 Department of Advanced Ophthalmic Imaging, Institute of Science Tokyo, Tokyo 113-8519, Japan

**Keywords:** iStent inject W, high myopia, open-angle glaucoma, minimally invasive glaucoma surgery

## Abstract

**Background**: The purpose of this study was to evaluate the 2-year postoperative outcomes of iStent inject W implantation combined with phacoemulsification, with a specific focus on comparing results between high myopia (HM) and non-high myopia (non-HM) eyes. **Methods**: In this retrospective study, subjects were divided into HM and non-HM groups using an axial length cut-off of 26.0 mm. The main outcome measures were intraocular pressure (IOP), number of glaucoma medications, surgical success rates, and postoperative complications. Surgical success was defined as a decrease in glaucoma medical therapy of ≥1 medication without an increase in IOP, or a postoperative IOP ≤ 21 mmHg with a ≥20% reduction. Changes from baseline were analyzed up to 24 months postoperatively. **Results**: The non-HM group achieved a significant IOP reduction at all postoperative time points. The HM group showed no significant postoperative IOP reduction except at 3 months. The mean number of medications significantly decreased from baseline to 24 months in two groups. The cumulative surgical success rate at 24 months was 48.6% in the non-HM group, whereas it was significantly lower at 19.4% in the HM group (*p* = 0.038). No significant difference in the incidence of postoperative complications was observed between the groups. **Conclusions**: The IOP-lowering efficacy of iStent inject W implantation combined with phacoemulsification appeared limited in eyes with HM. These results may suggest the need for careful patient selection and clinical judgment when considering iStent inject W implantation in this population.

## 1. Introduction

Glaucoma is one of the leading causes of irreversible blindness worldwide [1] with the number of affected individuals projected to increase to 111.8 million by 2040 [2]. Lowering intraocular pressure (IOP), either through medications or surgical interventions, is the only proven strategy to slow the progression of visual field loss [3,4]. In recent years, the surgical options for mild to moderate glaucoma have expanded with the introduction of minimally invasive glaucoma surgery (MIGS) [5,6]. Among these, the iStent trabecular micro-bypass was the first stent-based MIGS device to receive approval from the U.S. Food and Drug Administration (FDA) [7]. A second-generation device, the iStent inject W (Glaukos Corporation, San Clemente, CA), incorporates two stents designed to enhance aqueous outflow through the trabecular meshwork (TM) and is most commonly implanted in combination with cataract surgery [8,9,10,11]. Registry analyses confirm the rapid and widespread adoption of iStent procedures in clinical practice [12].

High myopia (HM), commonly defined as axial length (AL) ≥ 26.0 mm, has become a critical ophthalmic concern due to its rising prevalence; it is projected that approximately 10% of the world’s population will be affected by HM by 2050 [13]. HM is associated with serious complications, such as retinal detachment, macular changes, choroidal atrophy, and open-angle glaucoma (OAG) [13,14,15]. Previous epidemiological studies have reported an association between HM and OAG, suggesting that patients with HM may be at increased risk of glaucomatous damage [16,17,18,19]. Although the clinical use of MIGS has expanded as the number of patients with HM increases, evidence regarding the surgical outcomes of MIGS procedures in highly myopic eyes remains limited [20,21,22,23]. In particular, there are few reports on the long-term efficacy and safety of iStent inject W implantation in this population.

The present study aimed to evaluate the 24-month outcomes of iStent inject W implantation combined with cataract surgery in HM eyes with OAG. We assessed postoperative IOP reduction, medication use, and safety and compared outcomes between HM and non-HM eyes.

## 2. Materials and Methods

### 2.1. Patients

This was a retrospective, real-world study conducted at the Institute of Science Tokyo Hospital. The study protocol received approval from the Institutional Review Board of the Institute of Science Tokyo Hospital (Study Approval Number: M2016-030). All participants provided written informed consent, and the study adhered to the ethical principles of the Declaration of Helsinki.

This retrospective study involved a chart review of 143 eyes from 143 participants who underwent iStent inject W implantation in combination with phacoemulsification at the Institute of Science Tokyo Hospital between 1 March 2020, and 31 October 2023. Inclusion criteria encompassed mild to advanced OAG diagnosed according to the American Academy of Ophthalmology (AAO) Preferred Practice Pattern and European Glaucoma Society (EGS) Guidelines [24,25]. Participants were categorized into two groups based on AL: the non-HM group (AL < 26.0 mm) and the HM group (AL ≥ 26.0 mm). Exclusion criteria comprised eyes undergoing another procedure during the operation, eyes with angle-closure glaucoma or secondary glaucoma (such as pseudoexfoliative glaucoma, pigmentary glaucoma, or neovascular glaucoma), and eyes with a history of laser therapy or previous intraocular surgery, including glaucoma surgery or vitrectomy. To avoid inter-eye correlation and potential confounding, only one eye per patient was included; in cases in which both eyes underwent surgery, the first-operated eye was selected. Because this was a retrospective, single-center study, some eyes were lost to follow-up, and eyes with incomplete data at each postoperative time point were excluded from the analyses for that time point.

### 2.2. Outcome Measures

Baseline data were collected at the final preoperative visit, and postoperative data were gathered at 1, 3, 6, 9, 12, 15, 18, 21, and 24 months. The 24-month follow-up was selected to align with recommended outcome reporting for MIGS studies, which includes evaluation of 2-year cumulative surgical success using Kaplan–Meier analysis [26]. Preoperative evaluation and data collection included comprehensive ophthalmic history, Best-Corrected Visual Acuity (BCVA), number of glaucoma medications, central corneal thickness (CCT), AL, mean deviation (MD) calculated from the Humphrey Field Analyzer (HFA) 30-2 program using the Swedish Interactive Threshold Algorithm (SITA), and IOP measurements. Decimal visual acuity was converted to the logarithm of the minimum angle of resolution (logMAR) for statistical analysis. IOP was measured using Goldmann applanation tonometry (Haag-Streit, Bern, Switzerland). Baseline IOP represents medicated IOP, as no preoperative medication washout was performed. The main outcome measures were IOP, number of glaucoma medications, surgical success probability determined by survival analysis, and postoperative complications. Comparisons with preoperative baseline values were performed for IOP and number of glaucoma medications within each group. Furthermore, between-group comparisons were conducted for percentage IOP reduction at 12 and 24 months, cumulative surgical success probability, and the incidence of postoperative complications. Surgical success was defined according to the American Academy of Ophthalmology Glaucoma Preferred Practice Pattern criteria as meeting either of the following: a decrease in glaucoma medical therapy of ≥1 medication from baseline without an increase in IOP, or a postoperative IOP ≤ 21 mmHg with a ≥20% reduction from baseline. Success required the absence of an increase in glaucoma medications, additional laser or incisional glaucoma surgery, loss of light perception vision, and hypotony (IOP ≤ 5 mmHg). Treatment failure was defined as inadequate IOP control (IOP > 21 mmHg or <20% reduction from baseline) or hypotony present on two consecutive postoperative visits [26]. Because IOP and glaucoma medication use are often unstable during the first 3 months after surgery, surgical success and failure were evaluated only at postoperative visits more than 3 months after surgery; eyes that required additional glaucoma surgery within the first 3 months were classified as failures. The incidence of postoperative adverse events, including transient IOP elevation, hyphema, prolonged inflammation, macular edema, hypotony, endophthalmitis, and rhegmatogenous retinal detachment, was compared between the non-HM and HM groups. Transient IOP elevation was defined as an increase of ≥10 mmHg from baseline or an absolute IOP of ≥30 mmHg occurring within 1 month postoperatively. Postoperative hyphema was defined as clinically significant layered hyphema observed in the anterior chamber at any visit during the first postoperative month. Therefore, given the expected blood reflux following Schlemm’s canal (SC) surgery, mild hyphema or reflux not requiring specific intervention was not categorized as an adverse event. To further assess safety, the need for additional glaucoma surgery during the 24-month follow-up period was compared between the groups.

### 2.3. Surgery and Postoperative Management

All surgeries were conducted by two experienced surgeons (TY and SY). Phacoemulsification was performed as the initial step in a standardized routine manner, followed by the implantation of a one-piece acrylic intraocular lens into the capsular bag. The anterior chamber was filled with cohesive viscoelastic, and the patient’s head was turned away from the surgeon while the operating microscope was tilted at a 45-degree angle. A gonioprism was placed on the cornea to ensure an optimal view of the angle. For iStent inject W implantation, the stents were nasally implanted into the TM at an angle of 30–60 degrees. No viscoelastic was left in the anterior chamber, and the IOP was maintained in the mid-20s to minimize blood reflux. Postoperatively, patients received topical therapy, including 0.1% dexamethasone (Sanbetazone, Santen, Tokyo, Japan) and 1.5% levofloxacin (Cravit, Santen), four times daily for 3 weeks, with a tapering schedule based on the resolution of inflammation. IOP level, glaucoma medication use, and complications were recorded at 1, 3, 6, 9, 12, 15, 18, 21, and 24 months postoperatively. Additional glaucoma medications or discontinuation of existing ones were determined postoperatively based on the surgeon’s clinical judgment, considering disease severity and target IOP. This decision-making process aligned with the AAO Preferred Practice Pattern [24] and the EGS guidelines [25].

### 2.4. Data Analysis

All data were anonymized before statistical analysis. Analyses were performed using RStudio (version 2025.09.2, build 418; RStudio Team, Boston, MA, USA), a graphical user interface for R (version 4.4.2; The R Foundation for Statistical Computing, Vienna, Austria).

Continuous variables are presented as mean ± standard deviation (SD). Between-group comparisons were performed using the Mann–Whitney U test for continuous variables (including baseline characteristics, absolute IOP reduction, and percentage IOP reduction). Categorical variables, including sex and the incidence of postoperative complications, were compared using Fisher’s exact test. Longitudinal changes in IOP and the number of glaucoma medications were analyzed using the Wilcoxon signed-rank test. To account for multiple comparisons in these longitudinal analyses, *p*-values were adjusted using the Benjamini–Hochberg false discovery rate procedure. Survival analyses were performed using the Kaplan–Meier method, with intergroup differences assessed using the log-rank test. Additionally, Cox proportional hazards regression was used to explore potential prognostic factors. For the multivariable Cox regression analysis, covariates were restricted to key prognostic factors (age, preoperative IOP, medication count, and BCVA) to ensure model stability given the number of observed failure events. Fixed-combination glaucoma medications were counted as two separate drugs. All statistical tests were two-tailed, and statistical significance was defined as an adjusted *p*-value < 0.05.

## 3. Results

### 3.1. Patient Demographics and Baseline Characteristics

A total of 143 eyes from 143 patients were included. The mean age was 71.7 ± 10.4 years, and the cohort consisted of 64 men (44.8%) and 79 women. The overall baseline IOP was 15.8 ± 4.5 mmHg, and patients were receiving an average of 2.7 ± 1.5 glaucoma medications. The mean BCVA was 0.3 ± 0.3 logMAR, and the mean visual field MD was −12.1 ± 7.2 dB. The mean CCT was 520.4 ± 38.9 µm, and the mean AL was 26.0 ± 2.8 mm, as shown in Table 1.

For the subgroup analysis, 83 eyes were categorized as non-HM and 60 eyes as HM. The HM group was significantly younger than the non-HM group (65.6 ± 10.2 vs. 76.1 ± 8.1 years, *p* < 0.001) and had a longer AL (28.8 ± 2.1 vs. 24.0 ± 1.0 mm, *p* < 0.001). The HM group used fewer glaucoma medications preoperatively (2.3 ± 1.4 vs. 2.9 ± 1.6, *p* = 0.016). BCVA was also significantly worse in the HM group than in the non-HM group (0.4 ± 0.3 vs. 0.2 ± 0.3, *p* = 0.002), whereas baseline IOP, CCT, and visual field MD were comparable between the two groups.

### 3.2. Efficacy Outcomes

Table 2 shows the time course of IOP over 24 months. In the overall cohort, mean IOP significantly decreased from 15.8 ± 4.5 mmHg at baseline to 13.5 ± 3.2 mmHg at 12 months (*p* < 0.001) and to 14.3 ± 4.3 mmHg at 24 months (*p* = 0.030). In subgroup analyses, the non-HM group showed a sustained reduction from 16.2 ± 4.3 mmHg at baseline to 13.1 ± 3.1 mmHg at 12 months (*p* < 0.001) and 14.2 ± 4.8 mmHg at 24 months (*p* = 0.010), whereas the HM group showed no significant change from baseline (15.1 ± 4.6 mmHg) at either 12 months (14.0 ± 3.3 mmHg, *p* = 0.428) or 24 months (14.5 ± 3.7 mmHg, *p* = 0.987).

Table 3 summarizes the time course of glaucoma medication use. In the overall cohort, the mean number of medications decreased from 2.7 ± 1.5 at baseline to 1.6 ± 1.5 at 12 months (*p* < 0.001) and remained reduced at 2.1 ± 1.6 at 24 months (*p* < 0.001). In subgroup analyses, both groups showed significant reductions compared with baseline. In the non-HM group, the mean number of medications decreased from 2.9 ± 1.6 at baseline to 1.7 ± 1.6 at 12 months (*p* < 0.001) and 2.2 ± 1.7 at 24 months (*p* = 0.005). Similarly, in the HM group, the medication count decreased from 2.3 ± 1.4 at baseline to 1.4 ± 1.3 at 12 months (*p* = 0.001) and 2.1 ± 1.5 at 24 months (*p* = 0.031).

**Figure 1:**
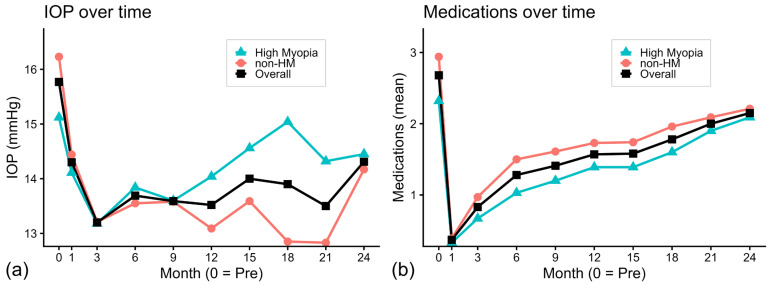
Longitudinal changes in intraocular pressure and medication use over 24 months. (**a**) Mean intraocular pressure (IOP, mmHg) and (**b**) mean number of topical glaucoma medications are plotted at baseline (0 = preoperative) and postoperative months 1, 3, 6, 9, 12, 15, 18, 21, and 24. The overall cohort is shown in black (squares), the non-high myopia (non-HM) group in red (circles), and the high myopia (HM) group in blue (triangles). Data points represent mean values, with sample sizes varying at each time point because of follow-up availability.

As shown in Figure 1a, the mean IOP decreased sharply after surgery, reaching its lowest level at month 3 in the overall cohort. After this point, IOP levels increased slightly but remained controlled. This pattern was evident in the non-HM subgroup, which maintained lower IOP levels throughout follow-up. In contrast, the HM subgroup showed a rebound after month 3, returning close to baseline levels in the later postoperative period.

Figure 1b shows changes in glaucoma medication use. In both groups, there was a substantial reduction in the number of medications immediately after surgery. Although the medication count gradually increased over time, it remained consistently lower than the preoperative baseline through 24 months in the HM and non-HM groups.

Table 4 summarizes absolute (ΔIOP) and percentage (%) changes in IOP from baseline. For the overall cohort, significant absolute IOP reductions were observed: 2.2 ± 3.3 mmHg at 12 months and 1.4 ± 4.8 mmHg at 24 months, corresponding to mean percentage reductions of 11.4 ± 19.9% and 5.5 ± 27.9%, respectively. In the non-HM group, significant and larger IOP reductions were observed (ΔIOP 3.3 ± 3.2 mmHg at 12 months and 2.7 ± 5.6 mmHg at 24 months), with mean percentage reductions of 18.2 ± 16.4% at 12 months and 12.9 ± 28.4% at 24 months. In contrast, the HM group showed only small, non-significant changes: ΔIOP was 0.8 ± 3.0 mmHg at 12 months and 0.1 ± 3.4 mmHg at 24 months, with mean percentage reductions of 3.3 ± 20.9% at 12 months and −2.3 ± 25.7% at 24 months. Percentage IOP reduction was significantly greater in the non-HM group at 12 months (18.2 ± 16.4% vs. 3.3 ± 20.9%, *p* = 0.003) and remained significant at 24 months (12.9 ± 28.4% vs. −2.3 ± 25.7%, *p* = 0.023).

**Figure 2:**
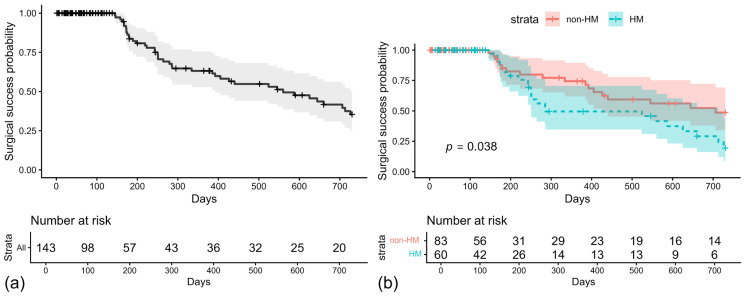
Kaplan–Meier survival curves for surgical success. (**a**) Cumulative probability of success for the overall cohort (black line) and (**b**) comparison between the non-high myopia (non-HM; red line) and high myopia (HM; blue line) groups. The log-rank test showed a significant difference between groups (*p* = 0.038). In both panels, vertical tick marks indicate censored cases, and the number of eyes at risk at each time point is listed below the *x*-axis. Analysis was truncated at 730 days.

Figure 2a shows the overall Kaplan–Meier curve for surgical success, with administrative censoring at day 730. The cumulative probability of success was 63.2% at 12 months and 35.4% at 24 months, and the median time to loss of success was 560 days. Figure 2b shows curves stratified by the group. The non-HM group had a significantly higher cumulative probability of success than the HM group (log-rank *p* = 0.038). At 12 months, the cumulative success probabilities were 74.4% in the non-HM group and 49.5% in the HM group. At 24 months, these rates decreased to 48.6% and 19.4%, respectively. The median time to loss of success was 706 days for the non-HM group and 286 days for the HM group. In a multivariable Cox proportional hazards model adjusting for age, preoperative IOP, preoperative medication count, BCVA, and HM status, none of the covariates were significantly associated with failure (Supplementary Table S1).

### 3.3. Safety and Adverse Events

Postoperative complications were infrequent and broadly comparable between groups (Table 5). Transient IOP elevation was the most common event, occurring in 6.0% of eyes in the non-HM group and 5.0% in the HM group, with no significant difference between groups. Regarding reinterventions, additional glaucoma surgery over 24 months was uncommon and did not differ significantly between groups. Overall, four eyes (2.8%) required further surgery, including three eyes (3.6%) in the non-HM group (two trabeculectomies and one micropulse transscleral cyclophotocoagulation) and one eye (1.7%) in the HM group (trabeculectomy). No cases of hyphema, prolonged inflammation, cystoid macular edema, hypotony, endophthalmitis, or choroidal complications were observed. One case of rhegmatogenous retinal detachment occurred in the HM group. Overall, the incidence of postoperative complications did not differ significantly between the two groups.

## 4. Discussion

In this study, we compared the outcomes of cataract surgery combined with iStent inject W implantation up to 24 months between patients with OAG with and without HM. In the entire cohort, significant reductions in IOP and the number of topical medications were sustained through 24 months. Similarly, in the non-HM group, IOP and the number of topical medications were significantly reduced, with sustained effects at 24 months. In contrast, surgical outcomes in the HM group were inferior than those in the non-HM group. In the HM group, except at the 3-month follow-up, no significant reduction in IOP from baseline was observed. However, the reduction in the number of topical medications persisted through 24 months. Surgical success was defined as the absence of additional laser treatment or glaucoma surgery, loss of light perception, or hypotony, with either of the following criteria met: a reduction of at least one glaucoma medication without an increase in IOP, or achievement of an IOP ≤21 mmHg with a ≥20% reduction from baseline while avoiding increases in medication number. Based on these criteria, the cumulative surgical success rate at 24 months was significantly lower in the HM group than in the non-HM group. This result suggests that the efficacy of iStent inject W implantation may differ in patients with HM and OAG than in patients without HM.

The prevalence of myopia is increasing globally. Although this trend is particularly evident in East Asia, rising rates have also been reported in other regions [27,28]. Since myopia, particularly HM, is a major risk factor for glaucoma, the incidence of glaucoma is expected to increase with the growing myopic population [29,30,31,32]. Consequently, opportunities for glaucoma surgery and iStent inject W implantation in myopic eyes, including those with HM, are expected to increase. Although previous reports have documented the outcomes of iStent inject W implantation combined with cataract surgery, most have focused on emmetropic to mildly myopic eyes, and no studies have specifically evaluated the longitudinal outcomes in patients with HM [8,9,10,11]. It has been reported that glaucoma surgical outcomes, including angle surgery, differ between HM and non-HM eyes in terms of IOP reduction and safety; however, such studies are limited in number [20,21,22,23]. Our recent analysis demonstrated that the target IOP reduction required for visual field preservation differs between HM and non-HM groups in filtration surgery [33]. Similarly, regarding standalone Kahook Dual Blade (KDB)-assisted trabeculotomy, the cumulative success rate at 36 months was significantly lower in the HM group (45%) than in the non-HM group (65%) [23]. With the widespread adoption of MIGS and the increasing use of iStent inject W, evaluating postoperative outcomes in HM eyes is critical for establishing tailored treatment approaches. To our knowledge, no study has directly compared the surgical outcomes of cataract surgery combined with iStent inject W between HM and non-HM groups in OAG eyes.

Long-term follow-up reports of iStent inject W are limited. In an analysis through 1 year postoperatively, Onoe et al. reported significant IOP and medication reduction in a group with a mean AL of 25.1 ± 2.1 mm undergoing cataract surgery combined with iStent inject W: IOP decreased from 15.0 ± 3.8 mmHg preoperatively to 13.2 ± 2.4 mmHg at 12 months (10.0%, *p* < 0.05), and medications decreased from 2.1 ± 1.4 to 0.5 ± 0.8 at 12 months (*p* < 0.05) [8]. Asaoka et al. also reported significant IOP and medication reduction in a group with mean AL of 24.7 ± 1.7 mm undergoing cataract surgery combined with iStent inject W: IOP decreased from 14.6 ± 3.3 mmHg preoperatively to 13.1 ± 2.4 mmHg at 12 months (10.3%, *p* < 0.001), and medications decreased from 1.6 ± 1.5 to 1.2 ± 1.2 at 12 months (*p* < 0.001) [9]. Regarding long-term surgical outcomes, Parrilla Vallejo et al. evaluated 3-year outcomes of cataract surgery combined with iStent inject W, reporting an IOP reduction from 15.9 mmHg preoperatively to 14.3 mmHg at 12 months (10.1%) and 14.3 mmHg at 24 months (9.9%). In the same report, medications were significantly reduced from 1.88 preoperatively to 0.45 at 12 months and 0.37 at 24 months (*p* < 0.01) [10]. Inatani et al. conducted a prospective study over 2 years following cataract surgery combined with iStent inject W, reporting significant IOP reduction from 15.9 mmHg to 13.2 mmHg at 12 months (approximately 17.0% reduction) and 13.7 mmHg at 24 months (approximately 13.3% reduction) (*p* < 0.001) [11]. In the same report, medications were significantly reduced from 2.4 preoperatively to 0.9 at 12 months and 1.0 at 24 months (*p* < 0.001). None of these reports included information regarding refractive status. Collectively, iStent inject W combined with cataract surgery has been reported to achieve approximately 10–17% IOP reduction at 12 months and 10–13% at 24 months, with medication reductions of approximately 0.5–1.5 agents at 12 months and approximately 1.5 agents at 24 months.

In our analysis, the non-HM group maintained favorable IOP control throughout the 24-month follow-up. IOP significantly decreased from 16.2 ± 4.3 mmHg preoperatively to 13.1 ± 3.1 mmHg (18.2 ± 16.4% reduction) at 12 months (*p* < 0.001) and to 14.2 ± 4.8 mmHg (12.9 ± 28.4% reduction) at 24 months (*p* = 0.010). The number of topical medications was also significantly reduced from 2.9 ± 1.6 preoperatively to 1.7 ± 1.6 at 12 months (*p* < 0.001) and 2.2 ± 1.7 at 24 months (*p* = 0.005). In the non-HM group, IOP reduction was comparable to that reported in previous studies. Although the baseline number of medications was slightly higher than in earlier reports, the reduction in medication burden remained substantial. In contrast, in the HM group, except at 3 months, no significant IOP change from baseline was observed. IOP changed from 15.1 ± 4.6 mmHg preoperatively to 14.0 ± 3.3 mmHg at 12 months (3.3 ± 20.9% change, *p* = 0.428) and to 14.5 ± 3.7 mmHg at 24 months (−2.3 ± 25.7% change, *p* = 0.892). However, the number of topical medications was significantly reduced from 2.3 ± 1.4 preoperatively to 1.4 ± 1.3 at 12 months (*p* = 0.001) and to 2.1 ± 1.5 at 24 months (*p* = 0.031). Although the HM group achieved a significant reduction in medication use, the extent of IOP reduction was more limited than that reported previously. Between-group comparisons revealed significant differences in the IOP reduction rate at 12 months (non-HM group, 18.2 ± 16.4% vs. HM group, 3.3 ± 20.9%, *p* = 0.003) and 24 months (non-HM group, 12.9 ± 28.4% vs. HM group, −2.3 ± 25.7%, *p* = 0.023). In the entire cohort, IOP significantly decreased from 15.8 ± 4.5 mmHg preoperatively to 13.5 ± 3.2 mmHg (11.4 ± 19.9% reduction) at 12 months (*p* < 0.001) and to 14.3 ± 4.3 mmHg (5.5 ± 27.9% reduction) at 24 months (*p* = 0.030). The number of topical medications significantly decreased from 2.7 ± 1.5 preoperatively to 1.6 ± 1.5 at 12 months (*p* < 0.001) and to 2.1 ± 1.6 at 24 months (*p* < 0.001). The overall IOP reduction was slightly lower than previously reported, suggesting that the limited efficacy observed in HM eyes attenuated the overall results. A similar trend was observed for surgical success rates. The cumulative success rate at 24 months was 48.6% in the non-HM group, whereas the HM group showed a significantly lower rate of 19.4% (*p* = 0.038). Influenced by the lower outcomes in the HM group, the cumulative success rate in the entire cohort was 35.4%. This relatively low overall outcome should be interpreted in light of the specific characteristics of our study population. Our cohort included relatively more severe cases than those in previous reports. In this study, baseline medication numbers in the entire cohort and non-HM group (2.7 ± 1.5 and 2.9 ± 1.6, respectively) were higher than those reported by Inatani et al. (2.4 ± 1.3) [11], and preoperative MD values (−12.1 ± 7.2 and −12.9 ± 7.8 vs. −7.6 ± 6.1) were worse. A large multicenter study of gonioscopy-assisted transluminal trabeculotomy identified higher preoperative medication burden as an independent predictor of surgical failure [34], whereas a study of KDB with phacoemulsification reported that patients with worse preoperative visual fields had a higher risk of surgical failure [35]. These differences in patient characteristics likely contributed to the lower success rates observed in the entire cohort. Overall, although the HM group showed significantly lower surgical success rates and more limited IOP reduction than the non-HM group, the medication reduction effect persisted through 24 months. This finding suggests that iStent inject W combined with phacoemulsification effectively reduces medication burden in HM eyes, even if the IOP-lowering effect is smaller than that observed in non-HM eyes.

It has been reported that the IOP-lowering effect of treatments targeting the conventional outflow pathway is smaller in HM eyes than in non-HM eyes. In pharmacological treatment, the IOP reduction following the addition of the ROCK inhibitor ripasudil was greater in the non-HM group than in the HM group at 4 and 12 weeks, with significantly smaller IOP reduction observed in the HM group [36]. In surgical treatment, as mentioned earlier, the 36-month cumulative success rate of KDB-assisted trabeculotomy alone was significantly lower in the HM group (45%) than in the non-HM group (65%) [23]. The iStent inject W is designed to bypass the TM and directly deliver aqueous humor into SC, acting on the conventional outflow pathway [37]. Consistent with the characteristics of treatments targeting this pathway, a similarly limited IOP-lowering effect was observed in the present study. In the conventional pathway, aqueous humor flows from the TM through SC and subsequently to collector channels before draining into episcleral veins [38,39]. Stenosis or obstruction at any point along this pathway, including the collector channels or episcleral veins, can restrict aqueous outflow and thereby contribute to IOP elevation. Although scleral thinning in myopic eyes is most prominent at the posterior pole, it also significantly affects the anterior sclera in proportion to the degree of myopia [40,41]. This process is thought to involve scleral remodeling associated with axial elongation, characterized by decreased collagen synthesis and increased degradation, leading to ultrastructural changes such as reduced collagen fibril diameter and diminished mechanical strength [40,41,42,43,44,45,46,47]. Although these scleral structural changes may affect the status of episcleral vessels, no studies have demonstrated a direct relationship between episcleral vessels and myopia. However, vascular loss in the retina and choroid adjacent to the sclera has been reported in HM eyes, which may indirectly suggest changes in episcleral vasculature [48,49,50]. These scleral alterations may influence the outcomes of surgeries targeting the conventional outflow pathway, including iStent inject W implantation. Another potential mechanism involves structural changes in the TM and SC. Previous studies have reported enlargement of SC diameter and area, along with thinning of the TM, in highly myopic eyes [51,52]. Given that the TM typically thickens with increased extracellular matrix deposition in active outflow regions and remains thin in inactive regions [53], these morphological findings suggest that the TM in myopic eyes may have reduced outflow capacity. Furthermore, enlargement of SC may reflect myopic scleral remodeling or a compensatory response to increased resistance distal to the canal. This implies that elevated downstream resistance may restrict aqueous outflow and limit surgical efficacy despite successful trabecular bypass. Owing to these structural changes, SC bypass by iStent inject W may not achieve sufficient outflow capacity in HM eyes. Therefore, identifying HM preoperatively is crucial for optimizing surgical planning and managing postoperative expectations. As the global prevalence of HM continues to rise, these findings provide valuable insights for future clinical practice.

In this study, postoperative adverse events were rare and primarily transient, and no sight-threatening complications were observed. The most frequent complication was transient IOP elevation, occurring in 6.0% of non-HM and 5.0% of HM eyes, with no significant difference between groups. These incidence rates are consistent with previous reports of iStent inject W combined with cataract surgery, which documented transient IOP elevation rates ranging from approximately 2.8% to 8.1%, including cases attributed to steroid response [8,9,10,11]. The need for additional glaucoma surgery was also low (3.6% in non-HM vs. 1.7% in HM), consistent with previously reported reoperation rates of 0–2.0% [9,11]. Additionally, no cases of postoperative hyphema were observed in our cohort. Prior studies have similarly reported a low incidence of hyphema (1.8–6.0%) [8,9]. Collectively, these findings suggest that iStent inject W implantation maintains a favorable safety profile in highly myopic eyes, comparable to that observed in non-myopic populations.

This study has several limitations. The retrospective and non-randomized nature of this study implies that potential biases cannot be ruled out. Most patients lost to follow-up were clinically stable and referred back to local primary care providers. Given that the need for additional surgery was comparable between the groups, the risk of significant differential attrition bias is considered low. Although our 24-month follow-up aligns with current reporting standards for MIGS [26], the chronic nature of glaucoma calls for longer-term, larger-scale prospective studies to provide more rigorous and definitive evidence. In addition, we did not perform quantitative assessments of anterior segment structures or fully address systemic medical histories and concomitant medications, which could potentially influence IOP. Finally, without a phacoemulsification-only control group, the individual contribution of the iStent inject W to IOP reduction cannot be distinguished from that of cataract surgery. Previous studies have suggested that HM status or AL do not significantly influence the extent of IOP reduction following cataract surgery [54,55]. Based on these findings, we consider that the influence of cataract surgery itself on the postoperative IOP comparison between groups was minimal in our current analysis. Despite these limitations, this study serves as a foundational reference for iStent inject W outcomes specifically in highly myopic eyes and provides clinical insights into the distinct postoperative course in this population.

## 5. Conclusions

In conclusion, this study demonstrated that the IOP-lowering effect of iStent inject W combined with phacoemulsification in HM eyes with glaucoma was significantly smaller than that observed in non-HM eyes. Therefore, HM eyes needing significant IOP reduction require careful surgical selection and comprehensive preoperative counseling to align patient expectations. Although IOP in HM eyes returned to baseline levels by 6 months postoperatively, a marked and sustained reduction in the number of glaucoma medications was observed. In this regard, for HM eyes with stable IOP where the primary clinical goal is to reduce the medication burden, combined phacoemulsification and iStent inject W implantation remains a favorable surgical indication. Consequently, the selection of combined cataract surgery and iStent inject W implantation should be based on a careful assessment of HM status and the establishment of preoperative target IOP goals. To further validate these findings and refine evidence-based management protocols, future long-term, larger-scale, randomized prospective studies are warranted.

## Figures and Tables

**Table 1:** Baseline characteristics of the study population.

Parameter	Overall	Non-HM	HM	*p*-Value
**Eyes**	143	83	60	-
**Age (years), mean ± SD**	71.7 ± 10.4	76.1 ± 8.1	65.6 ± 10.2	<0.001 *
**Sex, ***n*****				
**Men**	64	37	27	>0.999
**Women**	79	46	33	>0.999
**IOP (mmHg), mean ± SD**	15.8 ± 4.5	16.2 ± 4.3	15.1 ± 4.6	0.062
**Medications, mean ± SD**	2.7 ± 1.5	2.9 ± 1.6	2.3 ± 1.4	0.016 *
**BCVA, mean ± SD**	0.3 ± 0.3	0.2 ± 0.3	0.4 ± 0.3	0.002 *
**Visual field MD (dB), mean ± SD**	−12.1 ± 7.2	−12.9 ± 7.8	−11.2 ± 6.3	0.382
**CCT (µm), mean ± SD**	520.4 ± 38.9	518.9 ± 39.5	522.3 ± 38.2	0.380
**Axial length (mm), mean ± SD**	26.0 ± 2.8	24.0 ± 1.0	28.8 ± 2.1	<0.001 *

* Indicates statistical significance compared with the non-HM group. SD, standard deviation; HM, high myopia; IOP, intraocular pressure; BCVA, Best-Corrected Visual Acuity; MD, mean deviation; CCT, central corneal thickness.

**Table 2:** Mean IOP at each time point.

Time Point	Baseline	1 M	3 M	6 M	9 M	12 M	15 M	18 M	21 M	24 M
Overall (*n*)	15.8 ± 4.5 (143)	14.3 ± 3.8 (136)	13.2 ± 2.6 (111)	13.7 ± 3.4 (70)	13.6 ± 3.3 (58)	13.5 ± 3.2 (61)	14.0 ± 4.1 (38)	13.9 ± 3.5 (50)	13.5 ± 3.5 (42)	14.3 ± 4.3 (45)
*p* (vs. baseline)	N/A	<0.001 *	<0.001 *	0.002 *	<0.001 *	<0.001 *	0.002 *	0.002 *	0.002 *	0.030 *
non-HM (*n*)	16.2 ± 4.3 (83)	14.4 ± 3.9 (80)	13.2 ± 2.5 (61)	13.6 ± 3.2 (38)	13.6 ± 3.4 (33)	13.1 ± 3.1 (33)	13.6 ± 3.0 (22)	12.8 ± 2.7 (26)	12.8 ± 3.0 (23)	14.2 ± 4.8 (23)
*p* (vs. baseline)	N/A	<0.001 *	<0.001 *	<0.001 *	<0.001 *	<0.001 *	<0.001 *	<0.001 *	<0.001 *	0.010 *
HM (*n*)	15.1 ± 4.6 (60)	14.1 ± 3.8 (56)	13.2 ± 2.8 (50)	13.8 ± 3.7 (32)	13.6 ± 3.3 (25)	14.0 ± 3.3 (28)	14.6 ± 5.3 (16)	15.0 ± 4.0 (24)	14.3 ± 4.0 (19)	14.5 ± 3.7 (22)
*p* (vs. baseline)	N/A	0.428	0.015 *	0.987	0.428	0.428	0.987	0.987	0.987	0.987

* Indicates statistical significance compared with baseline. N/A, not applicable; HM, high myopia; IOP, intraocular pressure; M, months.

**Table 3:** Mean number of glaucoma medications at each time point.

Time Point	Baseline	1 M	3 M	6 M	9 M	12 M	15 M	18 M	21 M	24 M
Overall (*n*)	2.7 ± 1.5 (143)	0.4 ± 0.8 (142)	0.8 ± 1.3 (114)	1.3 ± 1.4 (72)	1.4 ± 1.4 (63)	1.6 ± 1.5 (61)	1.6 ± 1.5 (50)	1.8 ± 1.5 (51)	2.0 ± 1.6 (43)	2.1 ± 1.6 (47)
*p* (vs. baseline)	N/A	<0.001 *	<0.001 *	<0.001 *	<0.001 *	<0.001 *	<0.001 *	<0.001 *	<0.001 *	<0.001 *
non-HM (*n*)	2.9 ± 1.6 (83)	0.4 ± 0.9 (82)	1.0 ± 1.4 (63)	1.5 ± 1.5 (38)	1.6 ± 1.6 (33)	1.7 ± 1.6 (33)	1.7 ± 1.7 (27)	2.0 ± 1.6 (26)	2.1 ± 1.6 (23)	2.2 ± 1.7 (24)
*p* (vs. baseline)	N/A	<0.001 *	<0.001 *	<0.001 *	<0.001 *	<0.001 *	<0.001 *	0.002 *	0.005 *	0.005 *
HM (*n*)	2.3 ± 1.4 (60)	0.3 ± 0.8 (60)	0.7 ± 1.1 (51)	1.0 ± 1.3 (34)	1.2 ± 1.3 (30)	1.4 ± 1.3 (28)	1.4 ± 1.3 (23)	1.6 ± 1.4 (25)	1.9 ± 1.6 (20)	2.1 ± 1.5 (23)
*p* (vs. baseline)	N/A	<0.001 *	<0.001 *	<0.001 *	<0.001 *	0.001 *	0.006 *	0.004 *	0.035 *	0.031 *

* Indicates statistical significance compared with baseline. N/A, not applicable; HM, high myopia; M, months.

**Table 4:** IOP reduction rate at 12 and 24 months.

Metric	Time Point	Overall	Non-HM	HM	*p*-Value
ΔIOP (mmHg)	12 M	2.2 ± 3.3 (*n* = 61)	3.3 ± 3.2 (*n* = 33)	0.8 ± 3.0 (*n* = 28)	0.002 *
ΔIOP (mmHg)	24 M	1.4 ± 4.8 (*n* = 45)	2.7 ± 5.6 (*n* = 23)	0.1 ± 3.4 (*n* = 22)	0.020 *
Reduction (%)	12 M	11.4 ± 19.9 (*n* = 61)	18.2 ± 16.4 (*n* = 33)	3.3 ± 20.9 (*n* = 28)	0.003 *
Reduction (%)	24 M	5.5 ± 27.9 (*n* = 45)	12.9 ± 28.4 (*n* = 23)	−2.3 ± 25.7 (*n* = 22)	0.023 *

* Indicates statistical significance compared with the non-HM group. HM, high myopia; IOP, intraocular pressure; M, months.

**Table 5:** Postoperative complications.

Adverse Events	Overall, *n* (%)	Non-HM, *n* (%)	HM, *n* (%)	*p*-Value
IOP elevation	8 (5.6%)	5 (6.0%)	3 (5.0%)	>0.999
Hyphema	0 (0.0%)	0 (0.0%)	0 (0.0%)	N/A
Prolonged inflammation	0 (0.0%)	0 (0.0%)	0 (0.0%)	N/A
Macular edema	0 (0.0%)	0 (0.0%)	0 (0.0%)	N/A
Cyclodialysis cleft	0 (0.0%)	0 (0.0%)	0 (0.0%)	N/A
Hypotony	0 (0.0%)	0 (0.0%)	0 (0.0%)	N/A
Endophthalmitis	0 (0.0%)	0 (0.0%)	0 (0.0%)	N/A
Rhegmatogenous retinal detachment	1 (0.7%)	0 (0.0%)	1 (1.7%)	0.420
Additional glaucoma surgery	4 (2.8%)	3 (3.6%)	1 (1.7%)	0.639
Interventions		Trab × 2	Trab × 1	
		MTC × 1		

HM, high myopia; IOP, intraocular pressure; N/A, not applicable; Trab, trabeculectomy; MTC, micropulse transscleral cyclophotocoagulation.

## Data Availability

The data that support the findings of this study are not publicly available due to ethical and privacy restrictions but are available from the corresponding author upon reasonable request.

## Supplementary Materials

The following supporting information can be downloaded at: https://www.mdpi.com/article/10.3390/jcm15031265/s1, Table S1: Multivariable Cox proportional hazards model for time to surgical failure (up to 24 months).

## Abbreviations

IOP	Intraocular Pressure
MIGS	Minimally Invasive Glaucoma Surgery
FDA	U.S. Food and Drug Administration
HM	High Myopia
AL	Axial Length
OAG	Open-Angle Glaucoma
AAO	American Academy of Ophthalmology
EGS	European Glaucoma Society
BCVA	Best-Corrected Visual Acuity
CCT	Central Corneal Thickness
HFA	Humphrey Field Analyzer
SITA	Swedish Interactive Threshold Algorithm
SD	Standard Deviation
CI	Confidence Interval
HR	Hazard Ratio
MD	Mean Deviation
KDB	Kahook Dual Blade
TM	Trabecular Meshwork
SC	Schlemm’s Canal
