# Supplementary material for: Influence of High Myopia on Outcomes of iStent Inject W Implantation Combined with Cataract Surgery up to 24 Months of Follow-Up
Source: J Clin Med. 2026 Feb 5;15(3):1265. doi: 10.3390/jcm15031265 (PMC12897585; doi:10.3390/jcm15031265)
Supplement: Supplementary file 1 [file jcm-15-01265-s001.zip › jcm-4093598-supplementary.pdf]

Journal of
Clinical Medicine

Supplementary Table S1. Multivariable Cox proportional hazards model for time to surgical failure (up to 24 months)

Covariate	HR (95% CI)	p value
High myopia*	1.48 (0.65–3.38)	0.347
Preoperative IOP	0.96 (0.87–1.06)	0.448
Preoperative medications	0.95 (0.76–1.18)	0.635
Age	0.99 (0.95–1.03)	0.538
BCVA	1.08 (0.99–1.17)	0.075

* Reference category: non-HM (non-HM, n = 83; HM, n = 60). HRs are adjusted for all covariates listed in the table. Continuous covariates are expressed per 1-unit increase, except for BCVA, which is expressed per 0.1 logMAR increase. HR, adjusted hazard ratio; CI, confidence interval; HM, high myopia; IOP, intraocular pressure; BCVA, best-corrected visual acuity.

 http://doi.org/10.3390/jcm15031265
